# Retraction: Identification of Differentially Expressed Genes Related to Dehydration Resistance in a Highly Drought-Tolerant Pear, *Pyrus betulaefolia*, as through RNA-Seq

**DOI:** 10.1371/journal.pone.0304430

**Published:** 2024-05-22

**Authors:** The PLOS ONE Editors

Following the publication of this article [1], the corresponding author contacted PLOS requesting retraction of the article because the first author changed their field of study. This authorship issue raised to PLOS did not meet the journal’s retraction criteria, but in reviewing the case, additional scientific concerns were raised by a member of the *PLOS ONE* Editorial Board that warranted retraction of the article.

Specifically:

- The title of the article suggests that the study assesses differentially expressed genes comparing different drought-tolerant pears. However, the Materials and Methods section does not mention the germplasm. As there is no susceptible germplasm used in the study, the authors rely on literature to draw conclusions about expressed transcripts. With this lack of information about germplasm and experimental procedure, the statistical analysis is unclear and thus the results shown in the manuscript are less reliable.
- The study does not appear to report sufficient information about biological and /or technical replicates and the sampling used during the study. In the absence of these data, the reported statistical inference of the electrolytic leakage data is not meaningful.
- Although the study reports relative water content, net photosynthetic rate, stomatal conductance, and transpiration rate, the Materials and Methods section of the study does not report how these traits were measured.
- It is questionable whether the 6 hours of dehydration reported in the study is a sufficiently long period of drought to cause the significant damage in the cytoplasmic membrane reported in Fig 1.
- Multiple figures do not appear to report error bars.

In light of the above concerns, the *PLOS ONE* Editors retract this article.

All authors either did not respond directly or could not be reached.
